# Pilot study on novel blood containers with alternative plasticizers for red cell concentrate storage

**DOI:** 10.1371/journal.pone.0185737

**Published:** 2017-09-28

**Authors:** Yuki Morishita, Yusuke Nomura, Chie Fukui, Tsuyoshi Kawakami, Toshiyuki Ikeda, Tomokazu Mukai, Toshiyasu Yuba, Ken-ichi Inamura, Hisatoki Yamaoka, Ken-ichi Miyazaki, Hitoshi Okazaki, Yuji Haishima

**Affiliations:** 1 Division of Medical Devices, National Institute of Health Sciences, Setagaya, Tokyo, Japan; 2 Division of Environmental Chemistry, National Institute of Health Sciences, Setagaya, Tokyo, Japan; 3 Department of Transfusion Medicine, the University of Tokyo, Bunkyo, Tokyo, Japan; 4 Corporate Research and Development Division, Kawasumi Laboratories, Inc., Minato, Tokyo, Japan; 5 New Japan Chemical Co., Ltd., Chuo, Osaka, Japan; Hokkaido Daigaku, JAPAN

## Abstract

Di (2-ethylhexyl) phthalate (DEHP), a typical plasticizer used for polyvinyl chloride (PVC) blood containers, is eluted from the blood containers and exerts protective effects on red blood cells. However, a concern for detrimental effects of DEHP on human health has led to the development of potential DEHP substitutes. Here, we compared the red blood cell preservation ability of two types of non-DEHP blood containers with safe alternative plasticizers to that of DEHP blood containers. Red cell concentrates in mannitol-adenine-phosphate solution (MAP/RCC) were stored for 6 weeks in PVC blood bags containing DEHP, di-isononyl-cyclohexane-1,2-dicarboxylate (DINCH) and di (2-ethylhexyl) 4-cyclohexene-1,2-dicarboxylate (DOTH), or 4-cyclohexene-1,2-dicarboxylic acid dinonyl ester (DL9TH) and DOTH. There was no significant difference in the total amount of plasticizer eluted into MAP/RCC (till 3 weeks from the beginning of the experiment), hemolysis of MAP/RCC, and osmotic fragility of MAP/RCC between the non-DEHP blood containers and DEHP blood containers. Hematological and blood chemical indices of MAP/RCC in all containers were nearly the same. Thus, DOTH/DINCH and DOTH/DL9TH blood containers demonstrate the same quality of MAP/RCC storing as the DEHP blood containers. Since DOTH, DINCH, and DL9TH were reported to be safe, DOTH/DINCH and DOTH/DL9TH blood containers are promising candidate substitutes for DEHP blood containers.

## Introduction

Polyvinylchloride (PVC) has been widely used for the construction of medical devices, including blood containers, due to its durability and chemical inertness [1]. As it is inflexible, to produce medical devices, the use of plasticizers is required. Di (2-ethylhexyl) phthalate (DEHP) has been a typical plasticizer used for PVC-made medical devices [2, 3]. However, DEHP exhibits reproductive and developmental toxicity in rodents [4–7], while some studies reported the effects on human reproductive health [8, 9]. Therefore, concerns regarding the safety of DEHP led to stricter regulation of DEHP use in PVC products [10, 11] and the substitution of DEHP with alternative plasticizers [12, 13]. However, DEHP is eluted from the blood containers and it exerts beneficial effects on the storing of red blood cells, such as the decrease in hemolysis and increase in post-transfusion survival [14]. Because of this, the use of PVC blood bags containing DEHP has been permitted even in countries that are active for DEHP ban, such as Europe and Japan. Therefore, for the use in blood bags, alternative plasticizers should not only be safer, but also have protective effects on red blood cells comparable to those of DEHP.

Development and identification of suitable plasticizers for blood containers has been attempted previously. For example, we demonstrated that di (2-ethylhexyl) 4-cyclohexene-1,2-dicarboxylate (DOTH), diisodecyl phthalate, and di-isononyl-cyclohexane-1,2-dicarboxylate (DINCH, BASF SE, Germany) exhibit protective effects on red blood cells [15]. DINCH is one of the promising alternative plasticizers for PVC-made blood containers that has been commercially used [1], but with AS-1 as an additive solution, DINCH-PVC blood containers were reported to be inferior to DEHP-PVC blood containers for the prevention of hemolysis without mixing during storage [16]. DINCH elution into the blood product was shown to be significantly reduced, compared with that of DEHP, and during storage in saline-adenine-glucose-mannitol (SAGM) solution, the use of DINCH-based blood containers resulted in increased hemolysis compared with that observed in DEHP-based blood containers, although DINCH-based blood containers performed equivalently to DEHP-based containers with some alternative additive solutions [17]. Therefore, further improvement of blood containers with low toxicity and DEHP-equivalent protective effects on red blood cells should be achieved.

We recently reported that the concurrent use of DOTH and DINCH enabled the production of a safe PVC sheet with protective effects on red blood cells, which was comparable to that of DEHP [18]. Additionally, using a novel plasticizer, 4-cyclohexene-1,2-dicarboxylic acid dinonyl ester (DL9TH), we developed a PVC sheet for blood containers with comparable protective effects on red blood cells and improved cold resistance, compared to the characteristics of DEHP-PVC sheet [19]. Here, we compared the potential for red blood cell preservation in the DOTH/DINCH- and DOTH/DL9TH-PVC blood containers, and that in the DEHP blood containers.

## Materials and methods

### Ethics review

Experiments in this study were approved by the Ethics Committees of Kawasumi Laboratories, INC., The University of Tokyo Hospital, and National Institute of Health Sciences (approval numbers F6000, 11283, and 637–2, respectively). The procedure was performed in accordance with the ethical standards of the committees on human experimentation of Kawasumi Laboratories, INC., The University of Tokyo Hospital, and National Institute of Health Sciences. Written informed consents were obtained from all enrolled subjects.

### Materials

Two types of SANSO CIZER (DL9TH, Chemical Abstracts Service [CAS] number 1609185-22-9; and DOTH, CAS number 2915-49-3) were synthesized by New Japan Chemical Co., Ltd. (Osaka, Japan). Epoxidized soybean oil was purchased from Tokyo Chemical Industry Co., Ltd. (Tokyo, Japan). DINCH (CAS number 166412-78-8) was provided by BASF (Ludwigshafen, Germany), while DEHP (CAS number 117-81-7), DEHP-d4, diethyl ether of pesticide residue analysis grade, and phthalate-analytical-grade hexane were purchased from Kanto Chemical Co. (Tokyo, Japan). Sodium chloride from pesticide residue analysis grade, phthalate-analytical-grade anhydrous sodium sulfate (Wako Pure Chemical Industries, Ltd., Tokyo, Japan), and ultrapure water obtained using a Milli-Q Synthesis A10 system (Millipore, Tokyo, Japan) were used to prepare samples for gas chromatography/tandem mass spectroscopy (GC-MS/MS) analysis. Other chemicals were purchased from Wako Pure Chemical Industries. Prior to use, all tools made of glass, metal, or Teflon were heated to 250°C for more than 16 h.

### Preparation of blood containers

T-die molded PVC (degree of polymerization = 1,700) quadruple blood bag systems containing plasticizers (DEHP [55 parts to 100 parts PVC, w/w], DOTH/DINCH mixtures [25:33 parts to 100 parts PVC, w/w], or DOTH/DL9TH mixtures [25:33 parts to 100 parts PVC, w/w]), epoxidized soybean oil (8 parts to 100 parts PVC, w/w), and other additives were made according to a standard procedure of Kawasumi Laboratories, Inc. (Tokyo, Japan). The content of plasticizers in the blood bag systems was adjusted to obtain a plasticizing efficiency equivalent to that of the DEHP-containing blood bag systems.

### Whole blood collection and processing

Whole blood (400 mL) donated by 18 healthy volunteers was collected into DEHP-, DOTH/DINCH-, or DOTH/DL9TH-blood bag systems (n = 6, each) at The University of Tokyo Hospital. During collection, whole blood was mixed with 60 mL citrate-phosphate-dextrose solution. Whole blood samples were centrifuged (4200 ×*g*, 4°C, 8 min, slow deceleration) and plasma was separated. After separation of the buffy coat, 95 mL of mannitol-adenine-phosphate (MAP) solution (D-mannitol [14.57 g/L], adenine [0.14 g/L], sodium dihydrogen phosphate [0.94 g/L], sodium citrate hydrate [1.5 g/L], citric acid hydrate [0.2 g/L], glucose [7.21 g/L], and sodium chloride [4.97 g/L]) was added to the remaining red cell concentrates (RCCs) to prepare MAP/RCC. MAP/RCC was shipped at 4°C to the National Institute of Health Sciences for testing. MAP/RCC yield did not significantly differ between the analyzed blood containers (DEHP blood container, 277 ± 24 g; DOTH/DINCH blood container, 268 ± 16 g; and DOTH/DL9TH blood container, 276 ± 15 g).

### Sampling and storage

After mild mixing, MAP/RCC (7 mL) was collected under sterile conditions without air contact for testing at day 1 or 2 after processing, and thereafter weekly. MAP/RCC was stored at 4°C for 6 weeks.

### Plasticizer elution test

Plasticizer levels in MAP/RCC (after 0, 1, 2, 3, 4, 5, and 6 weeks of storage; n = 6) were determined using a previously described protocol, with some modifications [15, 19–23]. Briefly, aliquot (50 μL) of MAP/RCC was collected into screw-capped glass tubes, and sodium chloride (1 mL, 1 w/v%), DEHP-d4 (0.5 μg), and hexane (1 mL) were added. After vigorous shaking for 15 min and centrifuging at 3,000 rpm for 10 min at room temperature, the organic phase was collected and dehydrated with anhydrous sodium sulfate. Subsequently, the sample was analyzed by GC-MS/MS, which included an analysis of precision that was performed as described previously [15]. The retention times, the precursor (Q1) and product (Q2) ions of the plasticizers, and the collision energies of each plasticizer are presented in S1 Table. The product ions of all plasticizers were used for quantification, which was performed with DEHP-d4 as the internal standard. The concentrations of DINCH were determined using the sum of the total peak area of its isomers, as in a previous study [24]. The limits of detection and quantification (LOD and LOQ, respectively) were calculated using total optimization of chemical operations (TOCO) software version 2.0 and the function of mutual information (FUMI) theory [20]. The concentrations obtained using relative standard deviations of 33% and 10% based on the mass chromatograms of the standard and blank solutions, respectively, were used as the instrumental LOD and LOQ.

### Hemolysis test

The hemolysis test was performed after 0, 1, 2, 3, 4, 5, and 6 weeks of storage (n = 6), as described previously [19]. Briefly, an aliquot (50 μL) of MAP/RCC was collected into Eppendorf tubes (Eppendorf, Tokyo, Japan). Phosphate-buffered saline (1 mL) was added to each sample and gently mixed, followed by centrifugation at 425 ×*g* for 2 min at 4°C. Afterwards, the absorbance of the supernatant (100 μL) was measured at 415 nm with a SH-9000 Lab microplate reader (Corona Electric Co. Ltd., Ibaraki, Japan). Positive controls were prepared by adding distilled water instead of phosphate-buffered saline to the samples. The hemolytic ratio was calculated using the following formula: % hemolysis = 100 × AT/AP, where AT is the test sample absorbance and AP represents the average absorbance of the positive control.

### Osmotic fragility test

The osmotic fragility test was performed after 0, 3, and 6 weeks of storage (n = 6). Aliquots (10 μL) of MAP/RCC were collected into Eppendorf tubes. Serial dilutions of buffered salt solution (0.25, 0.35, 0.45, 0.55, 0.65, 0.75, 0.85, and 1.2% NaCl; 1 mL) were added to the samples and gently mixed. The mixtures were allowed to incubate for 30 min at 20°C and centrifuged at 425 ×*g* for 5 min. The absorbance of the supernatant (100 μL) was measured at 415 nm with a SH-9000 Lab microplate reader. Positive controls were prepared by adding distilled water instead of salt solution to the samples. The hemolytic ratio was calculated using the same formula as previously described.

### Hematological and blood chemical analyses

Hematological and blood chemical analyses of MAP/RCC (after 0, 1, 2, 3, 4, 5, and 6 weeks of storage; n = 6) were performed as follows. Concentration of adenosine triphosphate (ATP) and 2,3-diphosphoglycerate (2,3-DPG) were measured using ATP ASSAY kit for Blood (TOYO B-Net CO., LTD., Tokyo, Japan) and 2,3-DPG kit (Roche, Tokyo, Japan), respectively. Other hematological and blood chemical indices were measured by FALCO Biosystems Ltd. (Kyoto, Japan): red blood cell count was measured by the sheath flow DC detection method; hemoglobin levels were determined using the sodium lauryl sulfate hemoglobin method; hematocrit was measured by red blood cell pulse height detection method; mean corpuscular volume (MCV), mean corpuscular hemoglobin (MCH), and mean corpuscular hemoglobin concentration (MCHC) were calculated; total protein levels were measured by performing the biuret test; albumin levels were measured using the bromocresol purple method; the albumin/globulin ratio was calculated; glucose levels were determined by the hexokinase-glucose-6-phosphate dehydrogenase method; ammonia was measured by the modified Fujii-Okuda method; calcium was measured by the arsenazo III method; inorganic phosphorus levels were determined by the molybdic acid method; magnesium levels were measured by the Xylidyl Blue method; sodium, chloride, and potassium levels were measured by the electrode method.

### Statistical analysis

Data were analyzed by one-way analysis of variance (ANOVA) or two-way repeated measures ANOVA. A post hoc Tukey-Kramer test was performed on all ANOVA results that were shown to be significant.

## Results

### Plasticizer elution from blood containers

To examine the exposure to plasticizers, the amount of plasticizers eluted from blood containers into MAP/RCC (Table 1) was quantified. The quantities of plasticizer eluted from blood containers were shown to increase in a time-dependent manner for all plasticizers. Significant differences were found between the three types of blood containers using two-way repeated measures ANOVA (0–6 week). One-way ANOVA analysis followed by post hoc Tukey-Kramer test for values obtained each week demonstrated that the total amount of plasticizer eluted from the DOTH/DL9TH blood container was not significantly different compared with that eluted from the DEHP blood container, but the levels determined in the DOTH/DINCH group were significantly higher than those of the DEHP group at 4–6 weeks.

**Table 1:** Amount of plasticizer eluted from blood containers into MAP/RCC (μg/mL).

Blood containers	Measured plasticizers	0 week	1 week	2 week	3 week	4 week	5 week	6 week
DEHP	DEHP	2.6 ± 0.72^1)^	9.6 ± 4.6	12 ± 2.2	16 ± 2.6	17 ± 3.4	18 ± 4.8	22 ± 4.4
DOTH/DINCH	DOTH	2.2 ± 0.19	9.1 ±.3.9	12 ± 1.2	15 ± 2.5	18 ± 2.8	22 ± 6.4	26 ± 4.9
	DINCH	0.34 ± 0.01	1.8 ± 0.80	2.7 ± 0.18	3.9 ± 0.74	5.4 ± 0.44	7.0 ± 1.8	9.1 ± 1.8
	DOTH + DINCH	2.5 ± 0.20	11 ± 4.7	15 ± 1.3	19 ± 3.2	23 ± 3.2^2)^	29 ± 8.2^2)^	35 ± 6.6^3)^
DOTH/DL9TH	DOTH	2.5 ± 1.3	7.0 ± 2.6	8.9 ± 0.79	12 ± 2.3	16 ± 1.8	18 ± 2.1	21 ± 2.9
	DL9TH	0.43 ± 0.25	1.4 ± 0.62	2.1 ± 0.20	3.2 ± 0.72	4.4 ± 0.54	5.7 ± 0.77	6.9 ± 1.6
	DOTH + DL9TH	3.1 ± 1.5	7.3 ± 3.3	11 ± 0.87	16 ± 3.0	20 ± 2.1	23 ± 2.7	28 ± 4.2

^1)^Data are reported as mean ± SD (n = 6);

^2)^p < 0.05 vs. the DEHP group;

^3)^p < 0.01 vs. the DEHP group

### Hemolysis of stored MAP/RCC

**Fig 1:**
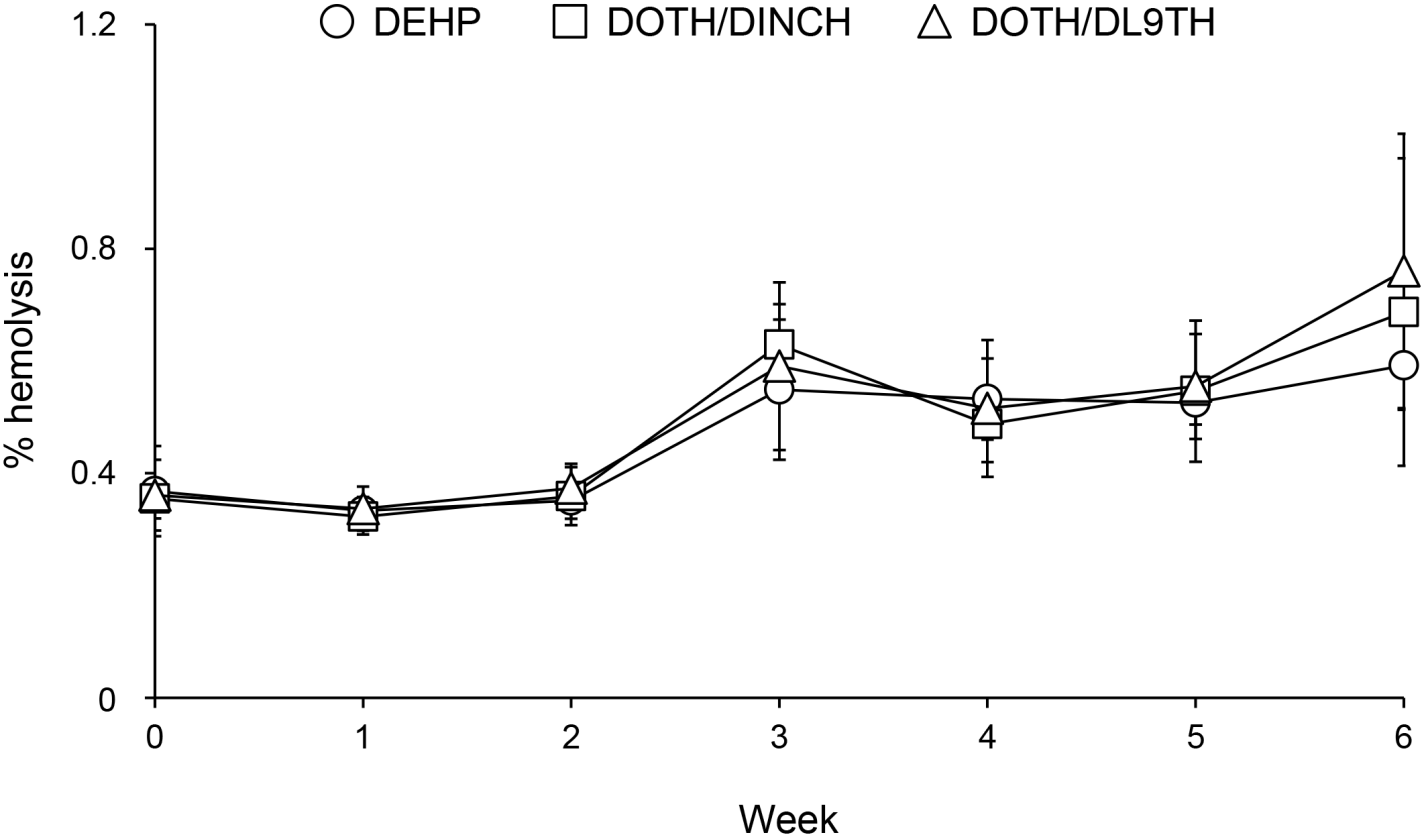
Hemolysis of stored MAP/RCC. Data are reported as mean ± standard deviation (SD; n = 6).

The hemolytic ratio of MAP/RCC did not significantly differ between the blood containers (Fig 1). The average hemolytic ratios at 6 weeks observed in the DEHP, DOTH/DINCH, and DOTH/DL9TH groups were 0.59, 0.69, and 0.76%, respectively.

### Osmotic fragility test

**Fig 2:**
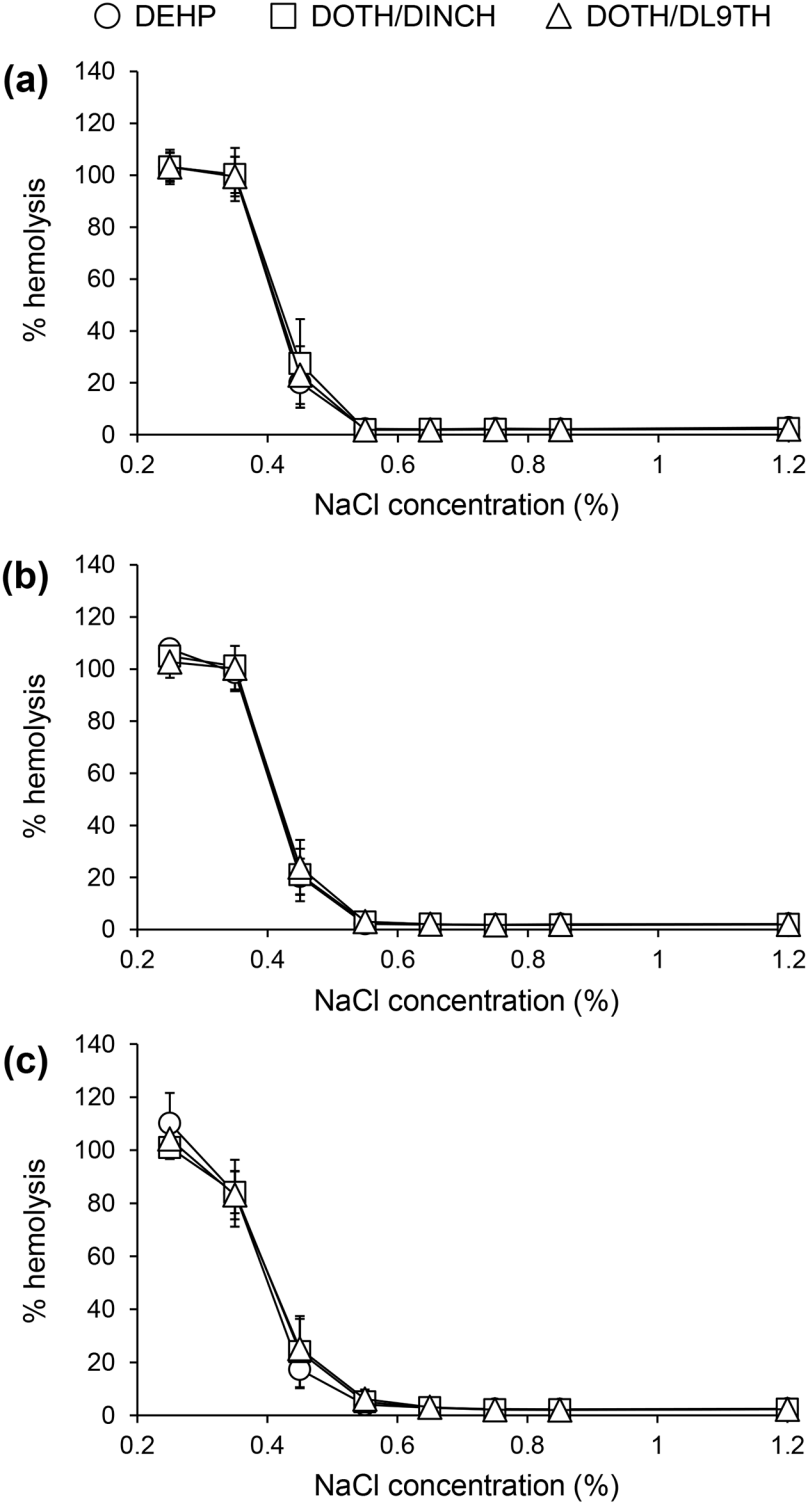
Osmotic fragility test of stored MAP/RCC. Hemolysis rate of MAP/RCC after 0 (a), 3 (b), and 6 weeks of storage (c) are shown. Data are reported as mean ± SD (n = 6).

In the osmotic fragility test (Fig 2), no considerable hemolysis was observed in 1.2, 0.85, 0.75, and 0.65% NaCl solutions, in all samples. In 0.55% NaCl, slight hemolysis was observed with the progression of storage time, while in 0.45, 0.35, and 0.25% NaCl solutions, considerable rate of hemolysis was observed, regardless of the storage period. The hemolytic ratio did not differ significantly between the blood containers.

### Hematological and blood chemical analyses

In hematological and blood chemical analyses of MAP/RCC (Table 2), significant differences were found between blood containers in MCV and MCH using two-way repeated measures ANOVA (0–6 weeks). These indices were higher in the DEHP group than in the other groups, although no significant differences were observed between groups by one-way ANOVA analysis of values obtained each week. The values were within the physiological range in all groups. Other than in MCV and MCH, no significant differences in hematological and blood chemical indices were observed by two-way repeated measures ANOVA analysis. However, one-way ANOVA analysis followed by a post hoc Tukey-Kramer test demonstrated that albumin, albumin/globulin ratio, and sodium levels were significantly higher in the DOTH/DINCH group than in the DEHP group at 6 weeks and that phosphorus level was significantly lower in the DOTH/DINCH and DOTH/DL9TH groups than in the DEHP group at 6 weeks.

**Table 2:** Hematological and blood chemical analyses of MAP/RCC.

Indices	Blood containers	0 week	1 week	2 week	3 week	4 week	5 week	6 week
Red blood cell count (×10^4^/μL)	DEHP	650 ± 41^1)^	740 ± 24	730 ± 91	740 ± 54	740 ± 85	700 ± 75	690 ± 71
	DOTH/DINCH	660 ± 36	760 ± 64	750 ± 90	730 ± 61	730 ± 130	720 ± 86	670 ± 59
	DOTH/DL9TH	690 ± 46	770 ± 45	790 ± 46	780 ± 55	770 ± 52	750 ± 80	700 ± 20
Hemoglobin (g/dL)	DEHP	21 ± 1.0	24 ± 1.2	23 ± 2.9	24 ± 1.4	23 ± 2.2	22 ± 1.1	22 ± 2.2
	DOTH/DINCH	20 ± 1.0	24 ± 2.1	24 ± 2.3	23 ± 2.2	23 ± 3.1	22 ± 2.5	21 ± 1.5
	DOTH/DL9TH	21 ± 1.6	24 ± 1.4	25 ± 1.7	24 ± 1.9	24 ± 1.9	23 ± 2.1	21 ± 0.89
Hematocrit (%)	DEHP	59 ± 1.9	69 ± 2.9	69 ± 7.5	72 ± 5.5	69 ± 7.2	65 ± 7.9	66 ± 6.5
	DOTH/DINCH	59 ± 1.9	70 ± 4.6	69 ± 5.6	67 ± 3.9	68 ± 8.8	67 ± 6.2	63 ± 3.9
	DOTH/DL9TH	60 ± 3.7	68 ± 3.7	71 ± 4.4	71 ± 5.6	70 ± 4.7	69 ± 9.3	64 ± 2.9
MCV (fL)	DEHP	92 ± 4.8	94 ± 4.0	95 ± 5.6	96 ± 4.8	94 ± 5.2	94 ± 6.4	95 ± 5.6
	DOTH/DINCH	90 ± 3.8	92 ± 3.9	92 ± 5.0	93 ± 4.3	93 ± 5.7	94 ± 5.3	94 ± 4.7
	DOTH/DL9TH	88 ± 3.1	89 ± 3.5	90 ± 3.7	91 ± 4.0	91 ± 3.3	92 ± 5.0	92 ± 5.1
MCH (pg)	DEHP	32 ± 1.8	32 ± 1.6	32 ± 2.1	32 ± 1.7	32 ± 1.8	32 ± 1.9	32 ± 1.6
	DOTH/DINCH	31 ± 1.1	31 ± 0.96	31 ± 1.2	31 ± 1.1	31 ± 1.4	31 ± 1.1	31 ± 1.0
	DOTH/DL9TH	31 ± 1.5	31 ± 1.4	31 ± 1.5	31 ± 1.4	31 ± 1.3	31 ± 1.7	31 ± 1.4
MCHC (%)	DEHP	35 ± 0.97	34 ± 0.55	34 ± 0.76	33 ± 1.4	34 ± 0.51	34 ± 0.57	34 ± 0.56
	DOTH/DINCH	35 ± 0.78	34 ± 1.0	34 ± 0.75	34 ± 0.81	34 ± 0.76	33 ± 0.94	33 ± 0.76
	DOTH/DL9TH	35 ± 0.81	35 ± 0.46	35 ± 0.29	34 ± 0.75	34 ± 0.57	34 ± 0.37	33 ± 0.68
Total protein (mg/dL)	DEHP	750 ± 250	750 ± 290	600 ± 140	780 ± 190	720 ± 260	750 ± 380	630 ± 120
	DOTH/DINCH	650 ± 190	830 ± 350	680 ± 170	750 ± 210	820 ± 390	770 ± 290	670 ± 100
	DOTH/DL9TH	630 ± 160	600 ± 170	770 ± 320	880 ± 550	750 ± 180	770 ± 360	670 ± 120
Albumin (mg/dL)	DEHP	300 ± 110	300 ± 60	270 ± 50	280 ± 80	250 ± 100	250 ± 80	200 ± 60
	DOTH/DINCH	330 ± 100	300 ± 130	280 ± 40	300 ± 130	320 ± 150	280 ± 80	300 ± 60^2)^
	DOTH/DL9TH	320 ± 40	280 ± 40	270 ± 80	300 ± 60	300 ± 60	270 ± 80	230 ± 50
Albumin/ globulin ratio	DEHP	0.68 ± 0.12	0.87 ± 0.48	0.85 ± 0.18	0.63 ± 0.27	0.63 ± 0.44	0.60 ± 0.24	0.47 ± 0.18
	DOTH/DINCH	1.4 ± 0.88	0.62 ± 0.26	0.78 ± 0.20	0.67 ± 0.29	0.72 ± 0.23	0.63 ± 0.14	0.85 ± 0.23^2)^
	DOTH/DL9TH	1.3 ± 0.88	1.3 ± 0.94	0.60 ± 0.19	0.70 ± 0.28	0.78 ± 0.40	0.67 ± 0.42	0.60 ± 0.25
ATP (μmol/g Hb)	DEHP	4.3 ± 0.48	5.2 ± 0.77	4.5 ± 0.55	4.1 ± 0.71	3.6 ± 0.62	2.6 ± 0.85	2.1 ± 0.59
	DOTH/DINCH	4.2 ± 0.39	4.8 ± 0.53	4.3 ± 0.29	4.1 ± 0.73	3.6 ± 0.41	2.6 ± 0.67	2.1 ± 0.47
	DOTH/DL9TH	4.4 ± 0.74	4.7 ± 0.66	4.7 ± 0.44	4.5 ± 0.58	4.1 ± 0.49	3.1 ± 0.49	2.6 ± 0.62
2,3-DPG (μmol/g Hb)	DEHP	42 ± 13	17 ± 12	1.4 ± 2.4	1.2 ± 1.9	0.38 ± 0.67	nd^4)^	1.5 ± 1.3
	DOTH/DINCH	35 ± 8.6	14 ± 9.1	0.95 ± 2.2	1.3 ± 2.1	0.61 ± 1.0	0.91 ± 1.5	1.4 ± 2.1
	DOTH/DL9TH	34 ± 7.8	19 ± 14	4.7 ± 4.8	nd	1.1 ± 2.7	0.71 ± 1.1	1.9 ± 1.8
Glucose (mg/dL)	DEHP	500 ± 21	420 ± 32	330 ± 46	230 ± 93	230 ± 54	200 ± 49	180 ± 53
	DOTH/DINCH	490 ± 18	410 ± 24	320 ± 36	280 ± 48	200 ± 88	160 ± 71	160 ± 56
	DOTH/DL9TH	490 ± 24	400 ± 27	310 ± 32	240 ± 44	150 ± 60	141 ± 63	120 ± 54
Ammonia (μg/dL)	DEHP	92 ± 11	190 ± 24	320 ± 33	460 ± 100	650 ± 140	790 ± 110	1200 ± 100
	DOTH/DINCH	93 ± 15	200 ± 30	340 ± 50	480 ± 110	670 ± 160	830 ± 180	1200 ± 200
	DOTH/DL9TH	95 ± 19	200 ± 25	300 ± 38	430 ± 86	600 ± 180	700 ± 100	1100 ± 170
Sodium (mEq/L)	DEHP	110 ± 1.4	100 ± 2.6	100 ± 3.6	98 ± 7.0	92 ± 4.9	91 ± 8.9	83 ± 3.8
	DOTH/DINCH	110 ± 2.1	110 ± 4.2	100 ± 3.1	99 ± 4.2	97 ± 9.5	92 ± 1.6	88 ± 1.4^2)^
	DOTH/DL9TH	110 ± 1.0	100 ± 3.0	98 ± 3.3	94 ± 3.6	93 ± 3.5	88 ± 2.4	86 ± 2.1
Chloride (mEq/L)	DEHP	77 ± 1.5	78 ± 1.2	75 ± 1.0	73 ± 4.2	75 ± 3.1	77 ± 0.75	77 ± 1.5
	DOTH/DINCH	77 ± 1.0	78 ± 1.2	76 ± 1.2	75 ± 1.9	76 ± 2.9	76 ± 1.8	77 ± 1.3
	DOTH/DL9TH	77 ± 1.5	78 ± 1.9	77 ± 1.0	76 ± 1.7	75 ± 3.0	77 ± 0.84	77 ± 1.3
Potassium (mEq/L)	DEHP	6.2 ± 1.8	20 ± 2.9	29 ± 4.9	31 ± 7.3	38 ± 9.8	45 ± 4.9	46 ± 6.8
	DOTH/DINCH	5.8 ± 0.80	19 ± 4.0	26 ± 1.2	30 ± 6.1	37 ± 4.7	39 ± 5.6	43 ± 2.4
	DOTH/DL9TH	6.7 ± 1.6	21 ± 3.3	30 ± 3.2	37 ± 3.5	35 ± 11	44 ± 6.4	47 ± 4.2
Calcium (μg/dL)	DEHP	600 ± 140	600 ± 110	630 ± 140	530 ± 240	700 ± 150	700 ± 180	730 ± 180
	DOTH/DINCH	650 ± 200	630 ± 200	700 ± 270	830 ± 290	630 ± 140	720 ± 370	750 ± 270
	DOTH/DL9TH	600 ± 60	600 ± 60	580 ± 80	630 ± 100	550 ± 280	670 ± 200	650 ± 210
Phosphorus (mg/dL)	DEHP	14 ± 1.3	15 ± 1.2	19 ± 1.1	19 ± 4.3	21 ± 1.2	22 ± 1.3	23 ± 0.75
	DOTH/DINCH	14 ± 1.0	15 ± 0.70	18 ± 0.74	19 ± 0.85	18 ± 5.4	20 ± 2.6	21 ± 0.84^2)^
	DOTH/DL9TH	14 ± 1.6	14 ± 1.1	18 ± 0.94	20 ± 1.2	18 ± 3.7	21 ± 2.1	21 ± 1.1^3)^
Magnesium (μg/dL)	DEHP	180 ± 80	320 ± 40	300 ± 60	450 ± 100	480 ± 130	530 ± 150	530 ± 80
	DOTH/DINCH	180 ± 80	320 ± 80	370 ± 80	450 ± 50	500 ± 200	520 ± 130	570 ± 140
	DOTH/DL9TH	220 ± 120	280 ± 80	350 ± 80	420 ± 40	470 ± 80	480 ± 100	480 ± 120

^1)^Data are reported as mean ± SD (n = 6);

^2)^p < 0.05 vs. the DEHP group;

^3)^p < 0.01 vs. the DEHP group;

^4)^nd: not detected

## Discussion

In the present study, red blood cell preservation ability of DOTH/DINCH- and DOTH/DL9TH-blood containers was compared to that of DEHP-blood containers.

In the plasticizer elution test, the total amount of plasticizer eluted into MAP/RCC from the DOTH/DINCH and DOTH/DL9TH blood containers did not significantly differ compared with that eluted from the DEHP blood container during the first 3 weeks. In Japan, where MAP solution is used as an additive, the expiration date of stored MAP/RCC is 21 days after processing. Therefore, the DOTH/DINCH and DOTH/DL9TH blood container use is comparable to that of the DEHP blood container. Although the total amount of plasticizer eluted into MAP/RCC from the DOTH/DINCH blood container was significantly higher compared with that eluted from the DEHP blood container during the following 3 weeks, these levels were only 1.59-fold higher than those determined in the DEHP blood container at 6 weeks after processing. We previously determined that the no-observed-adverse-effect level (NOAEL) of DOTH is 300 mg/kg body weight/day, using male rats in a 90-day oral repeat dose toxicity study [18]. The NOAEL of DINCH in male rats in an equivalent study was reported to be 107 mg/kg body weight/day [25]. A 13-week oral repeat dose toxicity study reported that the NOAEL of DEHP in male rats was 3.7 mg/kg body weight/day, although the exact NOAEL of DEHP remains controversial [26, 27]. Considering the improved safety of DOTH and DINCH in comparison with that of DEHP, the observed 1.59-fold increase in plasticizer levels is believed to be sufficiently low to avoid adverse effects in patients. We previously determined that the NOAEL of DL9TH is 717 mg/kg body weight/day in male rats, in a 90-day oral repeat dose toxicity study [19]. Therefore, similar plasticizer exposure levels between DEHP and DOTH/DL9TH blood containers indicates that the DOTH/DL9TH blood containers are safer for use than the DEHP blood container.

Hemolysis test results demonstrated that the hemolytic ratio of MAP/RCC did not significantly differ between the analyzed blood containers, suggesting that the hemolysis suppression in DOTH/DINCH and DOTH/DL9TH blood containers is not inferior to that of DEHP blood container. The hemolysis rate in this study tended to be higher than in other studies [16, 17], despite the higher hemolysis suppression effect of MAP solution compared to that of SAGM solution [28]. In this study, MAP/RCC was not leukoreduced, and the hemolysis rate was evaluated by a simplified method. These may be the causes for the high hemolysis rate, and conventional data for pharmaceutical application should be acquired separately. Currently, effective additive solutions are either available or are under development [13]. Therefore, the use of superior additive solutions to the MAP solution also improves the hemolysis rate of the RCC stored in DOTH/DINCH-PVC and DOTH/DL9TH-PVC bags. In this study, we mixed the stored MAP/RCC weekly upon sampling, and mixing during storage was reported to reduce the hemolysis of red blood cells [16, 29, 30]. Determining the extent to which mixing affected the hemolysis in our experiments is a subject for future analysis. However, all blood containers were analyzed at the same experimental conditions. Thus, we consider that the red blood cell preservation ability of blood containers was adequately compared by this pilot study.

In hematological and blood chemical analyses, we observed significant differences between groups in MCV and MCH levels, and these indices were shown to be higher in the DEHP group compared with those in the other analyzed groups. However, the initial values (at week 0) determined in the DEHP group were higher than those in the other groups. Additionally, MCV and MHC levels remained relatively stable with time, and within physiological range in all groups. This demonstrates that the observed significant differences in MCV and MCH were due to the different initial values between the groups, and that DOTH/DINCH and DOTH/TL9TH blood containers did not negatively affect MCV and MCH levels. We also observed significantly higher albumin, albumin/globulin ratio, and sodium levels in the DOTH/DINCH, and lower phosphorus in the DOTH/DINCH and DOTH/DL9TH groups than in the DEHP group at 6 weeks. High albumin, albumin/globulin ratio, and sodium, and low phosphorus in MAP/RCC do not mean low quality of stored red blood cells. Rather, these differences may indicate improved quality of red blood cells stored in the DOTH/DINCH and DOTH/DL9TH blood containers than those in the DEHP container, because a decrease in sodium and an increase in potassium and phosphorus levels are generally observed during storage. The glucose level of the DOTH/DL9TH group tended to be lower than that of the DEHP group, especially at 4–6 weeks, although no significant difference was found between the groups. Other indices such as ATP concentration were almost similar between the DOTH/DL9TH and DEHP groups, suggesting that the slight decrease in glucose concentration was not severe enough to deteriorate the quality of the stored RCC. In addition, until 3 weeks (the expiration date in Japan), the glucose level remained nearly the same between the groups. Furthermore, the osmotic fragility of stored MAP/RCC did not significantly differ between different groups, showing that the quality of MAP/RCC stored in the DOTH/DINCH and DOTH/TL9TH blood containers was not inferior to that of MAP/RCC stored in the DEHP blood container.

In conclusion, the data presented here demonstrates that DOTH/DINCH and DOTH/DL9TH blood containers represent suitable candidates for the replacement of DEHP blood containers, since they show improved safety and similar quality and total plasticizer exposure, compared with the DEHP blood containers. Considering that DOTH/DL9TH-PVC shows greater flexibility at lower temperatures than DOTH/DINCH-PVC [19], the DOTH/DL9TH-blood container is more promising. Currently, our group is also evaluating the quality of RCC samples stored in SAGM solution, an additive used worldwide. A pivotal study using pooled RCC, quadruple blood bag systems with filters for the selective removal of leukocytes, and a conventional method for the evaluation of hemolysis will be performed for pharmaceutical application.

## Supporting information

S1 Table Retention times, precursor ions (Q1), product ions (Q2), collision energies, LODs, and LOQs of the target chemicals. ^1)^Calculated using TOCO software, version 2.0 (FUMI theory). The values shown correspond to the concentration in the injected solution; ^2)^Internal standard.
(DOCX)
