# Supplementary material for: Pilot study on novel blood containers with alternative plasticizers for red cell concentrate storage
Source: PLoS One. 2017 Sep 28;12(9):e0185737. doi: 10.1371/journal.pone.0185737 (PMC5619825; doi:10.1371/journal.pone.0185737)
Supplement: S1 Table — 1)Calculated using TOCO software, version 2.0 (FUMI theory). The values shown correspond to the concentration in the injected solution; 2)Internal standard. (DOCX) [file pone.0185737.s001.docx]

**S1 Table. Retention times, precursor ions (Q_1_), product ions (Q_2_), collision energies, LODs, and LOQs of the target chemicals**

| **Chemicals** | **Retention time**  **(min)** | **Q_1_**  **(m/z)** | **Q_2_**  **(m/z)** | **Collision energy**  **(V)** | **LOD**^1)^  **(ng/mL)** | **LOQ**^1)^  **(ng/mL)** |
| --- | --- | --- | --- | --- | --- | --- |
| DOTH | 14.83 | 170 | 124 | 7 | 0.12 | 0.39 |
| DEHP | 15.20 | 167 | 149 | 4 | 0.051 | 0.17 |
| DINCH | 15.70–17.20 | 155 | 109 | 5 | 0.45 | 1.5 |
| DL9TH | 17.21 | 250 | 124 | 7 | 0.031 | 0.10 |
| DEHP-d_4_^2)^ | 15.19 | 171 | 153 | 4 |  |  |
